# Atrial Fibrillation and Primary Cilia-Associated Genes: The Role of CEP68

**DOI:** 10.3390/ijms27031498

**Published:** 2026-02-03

**Authors:** Zhenyu Dong, Rushd F. M. Al-Shama, Nicoline W. E. van den Berg, Makiri Kawasaki, Marc M. Terpstra, Nerea Arrarte Terreros, Elise L. Hulsman, Aldo Jongejan, Rishi A. Arora, Wim Jan P. van Boven, Antoine H. G. Driessen, Connie R. Bezzina, Sean J. Jurgens, Joris R. de Groot

**Affiliations:** 1 Department of Clinical and Experimental Cardiology, Amsterdam University Medical Center, University of Amsterdam, 1105 AZ Amsterdam, The Netherlands; z.dong@amsterdamumc.nl (Z.D.);; 2 Amsterdam Cardiovascular Sciences, Heart Failure and Arrhythmias, 1105 AZ Amsterdam, The Netherlands; 3 Departments of Biomedical Engineering and Physics, Amsterdam University Medical Center, University of Amsterdam, 1105 AZ Amsterdam, The Netherlands; 4 Departments of Radiology and Nuclear Medicine, Amsterdam University Medical Center, University of Amsterdam, 1105 AZ Amsterdam, The Netherlands; 5 Departments of Epidemiology and Data Science, Amsterdam University Medical Center, University of Amsterdam, 1105 AZ Amsterdam, The Netherlands; 6 Feinberg Cardiovascular and Renal Research Institute, Northwestern University Feinberg School of Medicine, Chicago, IL 60611, USA; 7 Department of Cardiothoracic Surgery, Amsterdam University Medical Center, University of Amsterdam, 1105 AZ Amsterdam, The Netherlands

**Keywords:** mendelian randomization, primary cilia, atrial fibrillation

## Abstract

Recent studies have demonstrated that primary cilia not only play a role in cardiovascular development, but also in the progression of acquired heart disease. Their role in atrial fibrillation (AF) is incompletely understood. We hypothesize that there is a causal link between primary cilia genes and the occurrence of AF. We integrated AF GWAS data with various multi-omic datasets—including data on gene expression, DNA methylation, and protein expression quantitative trait loci (eQTL, mQTL, and pQTL)—from human left atrial appendage (LAA) tissues and blood. Genetic variants linked to primary cilia-related genes were used as instrumental variables to explore their causal links to AF, through summary-data-based Mendelian randomization (SMR) and Bayesian colocalization. Single-cell sequencing data were used to analyze the expression of the selected genes across different cell types. The mechanisms by which the selected genes exert their effects were explored using RNA sequencing data, clinical indicators, and immunohistochemical markers from 22 patients without AF from the PREDICT-AF cohort, and 21 patients with paroxysmal AF and 19 patients with persistent AF from the MARK-AF cohort. Through SMR analyses, we established significant associations between predicted *CEP68* expression and AF in both blood (OR 1.25; 95% CI 1.18–1.33; false discovery rate (FDR) = 1.81 × 10^−9^) and LAA tissue (OR 1.12; 95% CI 1.08–1.16; FDR = 6.18 × 10^−9^). Moreover, predicted methylation of *CEP68* showed an inverse relationship with AF risk (OR 0.87; 95% CI 0.84–0.90; FDR = 2.55 × 10^−15^). Colocalization results for *CEP68* in both blood and the LAA indicated strong evidence of a shared causal variant. Within single-cell data, compared to the control group, AF patients had higher levels of *CEP68* in fibroblasts (*p* = 0.046). In bulk RNA-seq data, *CEP68* expression showed no significant differences among the no AF, paroxysmal AF, and persistent AF groups. *CEP68* was positively correlated with the cardiac remodeling marker Thrombospondin-2 in 22 patients without AF from the PREDICT-AF cohort (r = 0.45, *p* = 0.03). In AF patients from the MARK-AF study, *CEP68* was also positively associated with LAVI (r = 0.34, *p* = 0.03). Collectively, our results support a model in which genetically predicted CEP68 regulation is linked to AF liability and is consistent with fibroblast activation and remodeling-related pathways as potential mediators.

## 1. Introduction

Primary cilia are present in many cell types and are crucial for regulating complex cellular signaling networks. In cardiovascular diseases, the significance of primary cilia has been gradually recognized [1]. In contrast to the earlier belief that primary cilia were only involved in heart development, recent studies have also explored their relationship with acquired heart diseases [1,2].

We previously showed that primary cilia are degraded in atrial tissue of patients with persistent atrial fibrillation (AF) [3]. Knockdown of the primary cilia-specific gene Intraflagellar Transport 88 (IFT88) has been shown to lead to fibrosis by promoting fibroblast proliferation and differentiation into α-smooth muscle actin-expressing myofibroblasts, while increasing the production of extracellular matrix proteins [4]. Conversely, blockade of the upstream regulator HDAC6 with an AURKA-selective blocker inhibits the degradation of primary cilia and reverses these profibrotic processes in isolated human fibroblasts. Knockdown of IFT88 has further been shown to increase extracellular matrix protein expression and reduce conduction velocity in cardiomyocyte-fibroblast co-cultures [4]. These findings suggest that primary cilia disruption leads to extracellular matrix (ECM) remodeling and arrhythmogenic conduction abnormalities. Contrasting this notion, work by Villalobos et al. showed that the presence rather than the degradation of primary cilia is also necessary for cardiac fibrosis [5]. This apparent discrepancy may be due to the multifunctionality of primary cilia; their function is not necessarily correlated with their detectability or length, and their function may differ across the spectrum of cell types. Instead, given that primary cilia can extend or retract within hours or even minutes, their functional mechanisms may be far more complex and nuanced than we previously thought [6]. In addition, system-level regulators (e.g., circadian rhythm and metabolic state) may further modulate cilia-mediated signaling, potentially contributing to the dynamic, cell type-dependent effects observed across fibrotic contexts [7,8]. The specific molecular mechanisms remain to be elucidated.

Genome-wide association studies (GWAS) examine the links between genetic variants and traits, enabling the identification of quantitative trait loci (QTLs) and disease risk loci [9]. Summary-data-based Mendelian randomization (SMR) builds on Mendelian randomization (MR) principles by using independent GWAS summary statistics and QTL data to highlight possible causal genes from GWAS data [9]. A key benefit of MR is its ability to reveal causal relationships between exposures and outcomes while minimizing the risk of confounding and reverse causation. The concept of ‘AF begets AF’ complicates our understanding of the causal pathways that underlie the arrhythmia [10]. Therefore, MR represents an appealing approach to studying the molecular mechanisms of AF. We hypothesize that genetically predicted expression of primary cilia-associated genes is causally linked to AF. Moreover, as molecular biomarkers are increasingly integrated into multimodal cardiovascular risk prediction, such genes may also have translational relevance.

## 2. Results

### 2.1. Summary-Data-Based Mendelian Randomization Analysis of Primary Cilia Genome-Wide cis-eQTLs and AF

**Figure 1:**
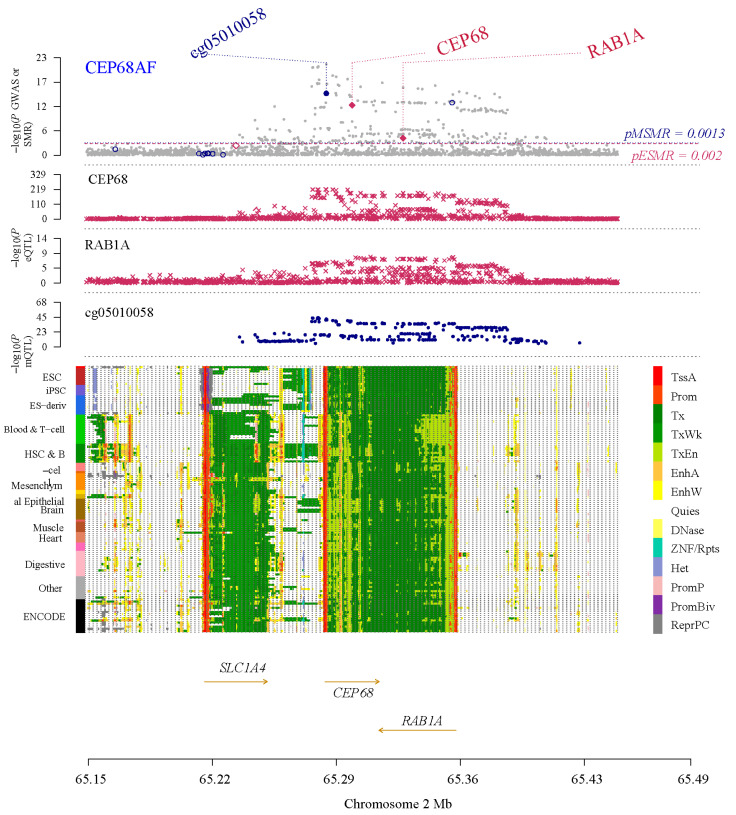
Results of SNP and SMR associations across mQTL, eQTL, and GWAS at the CEP68 locus, based on blood-derived expression data. The top plot shows −log10 (*p*-values) of SNPs from the AF GWAS. The red diamonds and blue circles represent −log10 (*p*-values) from SMR tests for associations of gene expression and methylation probes with AF, respectively. The second and third plots show eQTL results for the CEP68 and RAB1A. The fourth plot shows mQTL results for the methylation probe cg05010058 of CEP68. The bottom panel is a chromatin-state track across cell types; colors denote functional genomic states (such as promoter/enhancer/transcribed/quiescent), and arrows indicate gene orientation (transcriptional direction).

Within an SMR framework—integrating eQTL data with AF GWAS data—we identified primary cilia genes with expression causally linked to AF risk. Following SMR of blood-based expression, *ALB*, *ARRB2*, *CEP68*, *HNRNPF*, *NACA*, *PFKFB2*, *QPCT*, *REST*, *RPS2*, and *SF3B1* were significant and showed strong evidence of colocalization, indicating a potential causal link between their blood expression and AF. Separately, following SMR of left atrial appendage (LAA) expression, *CEP68*, *CCDC92*, *PFKFB2*, and *KDM1B* passed both SMR and colocalization, suggesting a potential causal link between their LAA expression and AF. Notably, CEP68 was identified in both the LAA and blood datasets, exhibiting a consistent trend across both (Figure 1). In the LAA set, the predicted *CEP68* expression showed an OR of 1.12 (95% CI 1.08–1.16; FDR = 6.18 × 10^−9^) and a colocalization probability of 0.86. In blood, predicted CEP68 expression showed a colocalization probability of 0.95, with an estimated OR of 1.25 (95% CI 1.18–1.33; FDR = 1.81 × 10^−9^), indicating that for each standard deviation increase in CEP68 expression, the risk of AF increased by approximately 25% (Figure S1A,B).

### 2.2. Summary-Data-Based Mendelian Randomization Analysis of Primary Cilia Genome-Wide cis-mQTLs and AF

In a similar framework, we assessed potential causal links between DNA methylation of primary cilia-related genes in blood and the risk of AF. Overall, 20 methylation sites were significant in SMR and showed strong colocalization evidence (Figure S2). Notably, the SNP rs2723064, associated with the *CEP68* gene and indexed by probe cg05010058, exhibited a significant inverse association with gene expression, with a protective effect on AF (OR = 0.87, 95% CI: 0.84–0.90), with robust statistical support (FDR = 2.55 × 10^−15^) and strong colocalization evidence (0.99) (Figure S2).

### 2.3. Summary-Data-Based Mendelian Randomization Analysis of Primary Cilia Genome-Wide cis-pQTLs and AF

We then assessed potentially causal links between protein abundance of primary cilia genes with AF, using SMR of pQTLs. In the SMR analysis of the blood proteome, RAB1A showed associations with AF, although colocalization analyses did not reach the 0.8 threshold (Figure S3). In LAA tissue, SMR suggested an inverse relationship between YBX3 with AF (FDR = 2.78 × 10^−2^), although the colocalization value of 0.09 indicated weak evidence of a shared causal variant (Figure S3).

### 2.4. Validation Using the FinnGen Consortium

In the replication phase, we utilized independent AF GWAS data from FinnGen, to validate the causal relationship between *CEP68* expression/methylation and AF. Using the FinnGen AF data, the SMR association between *CEP68* expression and AF was replicated using both blood (FDR = 8.18 × 10^−10^) and LAA (FDR = 6.62 × 10^−9^) eQTLs. Additionally, we examined the causal relationship between the *CEP68* methylation site cg05010058 and AF, which was also replicated (FDR = 2.39 × 10^−7^). It should be noted that this analysis is only a replication for the AF outcome data (which is independent of the AF GWAS data used in discovery), since the eQTL instruments were the same in discovery and replication.

### 2.5. The Steiger Method Analysis

We selected the *CEP68* gene for further study and scrutiny. We used the Steiger directionality test to assess whether *CEP68* expression/methylation leads to AF, rather than the other way around. The Steiger analysis demonstrated highly significant directional associations for *CEP68* using both blood (*p* = 1.92 × 10^−137^) and LAA (*p* = 4.88 × 10^−49^) eQTLs. These results suggest that the genetic regulation of *CEP68* expression may causally affect AF risk, rather than being a consequence of AF liability, in both tissue types. The Steiger method analysis also showed a highly significant directional association at the mQTL level for *CEP68* in blood (*p* = 1.61 × 10^−38^), suggesting that the changes in *CEP68* methylation may causally affect AF risk, rather than being a consequence of AF liability. Thus, our data support that the direction of effect is from CEP68 to AF (Figures S1 and S2).

### 2.6. Cell Type-Specific CEP68 Expression in Non-Cardiomyocytes in AF Versus Controls

**Figure 2:**
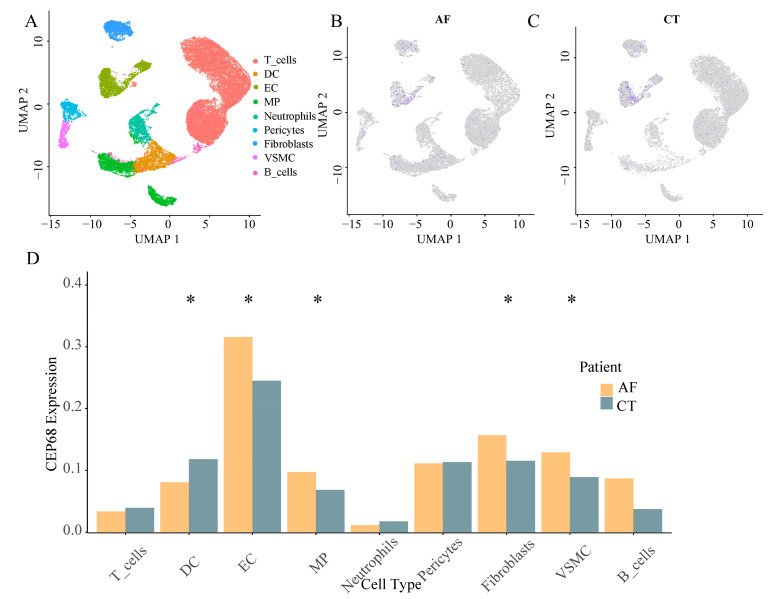
Single-cell type expression in left atrial tissue for CEP68. (**A**) A total of 9 cell types were identified. (**B**,**C**) Differences in CEP68 expression was observed across different cell types in both AF and control groups. Purple dots indicate higher CEP68 expression, whereas grey dots indicate low or undetected expression. (**D**) A comparison of CEP68 expression was made for each cell type between the AF and control groups. * indicates statistical significance between AF and control for the indicated cell type (*p* < 0.05). Abbreviations: AF, atrial fibrillation; CT, control; DC, dendritic cells; EC, endothelial cells; MP, macrophages; VSMC, vascular smooth muscle cells; UMAP, uniform manifold approximation and projection.

We identified nine major non-cardiomyocyte cell types using the analysis of single cell RNA sequencing, including vascular smooth muscle cells, T cells, endothelial cells, macrophages, fibroblasts, pericytes, neutrophils, B cells, and dendritic cells. We analyzed the differences in *CEP68* expression in these cell types between the AF and control groups. Among these, CEP68 expression in fibroblasts showed a modest increase in AF patients compared to the control group (*p* = 0.046). CEP68 expression was also significantly elevated in vascular smooth muscle cells, macrophages, and endothelial cells in the AF group. There were no differences in *CEP68* expression among T cells, pericytes, neutrophils and B cells between the AF and control groups (Figure 2).

### 2.7. Correlation and Gene Set Enrichment Analysis of CEP68 in Atrial Fibrosis

**Figure 3:**
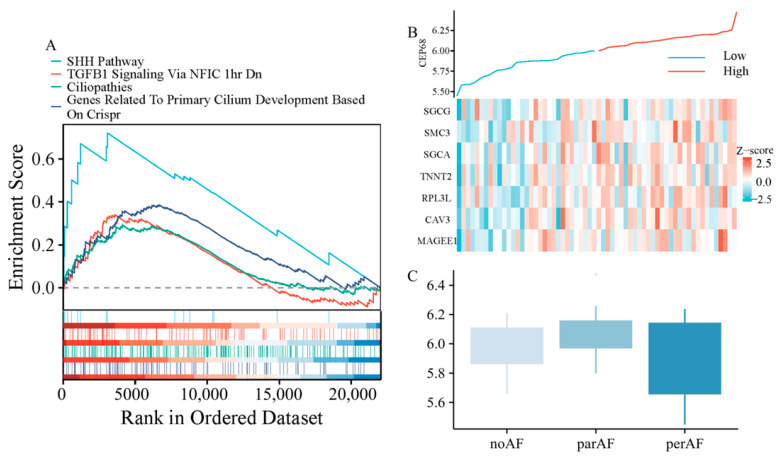
CEP68 Expression and Its Association with Fibrosis and Atrial Fibrillation. (**A**) GSEA demonstrates enrichment of fibrosis-related pathways and primary cilia-related pathways in relation to CEP68 expression. (**B**) Heatmap of atrial fibrosis-related gene expression ordered by CEP68 expression. Columns represent individual left atrial appendage samples (patients), ordered by increasing CEP68 expression from left to right; rows represent genes; colors indicate row-wise z-scored expression levels. (**C**) CEP68 expression did not differ significantly among the no AF, paroxysmal AF, and persistent AF groups.

We performed further analysis using RNA sequencing data from 62 patients, including those without AF, with paroxysmal AF, and with persistent AF. Gene set enrichment analysis (GSEA) analysis revealed that *CEP68* expression was significantly enriched in pathways associated with fibrosis, including TGF-β signaling-related pathways (normalized enrichment score NES = 1.54, FDR = 0.0085), as well as pathways associated with primary cilia, such as those involved in Sonic Hedgehog (SHH) signaling (NES = 2.13, FDR = 0.0004), cilium development (NES = 1.73, FDR = 0.0011), and ciliopathies (NES = 1.43, FDR = 0.0104) (Figure 3A).

In all 62 patients, we analyzed the correlation between CEP68 and genes associated with atrial fibrosis. The atrial fibrosis gene list was curated from GSEA pathways, including extracellular matrix components, interstitial cardiac fibrosis, and myocardial fibrosis. This analysis showed that *CEP68* expression was significantly associated with several atrial fibrosis-related genes (Figure 3B). For example, *CEP68* and *TNNT2* showed a positive correlation (r = 0.33, adjusted *p* = 0.002) after Bonferroni correction.

### 2.8. CEP68 Comparison Across Different Atrial Fibrillation Types

After adjusting for age and sex in RNA-seq data from all 62 patients, no significant difference in CEP68 expression was observed among the three groups—no AF, paroxysmal AF, and persistent AF (adjustedadjusted *p* = 0.106; Figure 3C). Similarly, in all 62 patients, no significant difference in CEP68 expression was found between AF (combining those with paroxysmal and persistent AF) and non-AF patients. Likewise, among the 22 patients without AF, CEP68 expression did not significantly differ between those who later developed AF and those who did not (adjusted *p* = 0.89).

### 2.9. Positive Correlation Between CEP68 and Atrial Remodeling Markers

**Figure 4:**
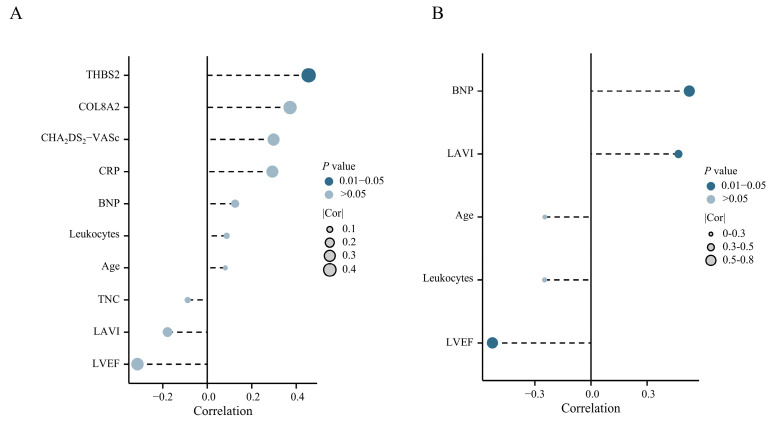
Clinical Correlation of CEP68. (**A**) Correlation analysis between CEP68 and clinical indicators in 22 no AF patients. (**B**) Correlation analysis between CEP68 and clinical indicators in 19 persistent AF patients. Abbreviations: AF, atrial fibrillation; BNP, B-type natriuretic peptide; CHA_2_DS_2_-VASc, Congestive heart failure, Hypertension, Age ≥ 75 (2 points), Diabetes mellitus, Stroke/TIA/thromboembolism (2 points), Vascular disease, Age 65–74, Sex category (female); COL8A2, collagen type VIII alpha 2; CRP, C-reactive protein; LAVI, left atrial volume index; LVEF, left ventricular ejection fraction; TNC, tenascin-C; THBS2, thrombospondin-2.

Among 22 patients without AF, *CEP68* expression showed a statistically significant correlation with Thrombospondin-2 (THBS2, r = 0.45, *p* = 0.03) (Figure 4A). In 40 patients with AF, *CEP68* expression showed a significant positive correlation with left atrial volume index (LAVI) (r = 0.34, *p* = 0.03). A similar association was observed in patients with persistent AF (r = 0.46, *p* = 0.044) (Figure 4B). Additionally, a positive correlation between CEP68 and N-terminal pro–B-type natriuretic peptide (NT-proBNP), as well as a negative correlation between *CEP68* and left ventricular ejection fraction (LVEF), was observed in patients with persistent AF (Figure 4B). Complete-case sensitivity analyses yielded similar results (Supplementary Figure S4). No other clinical indicators were significantly associated with CEP68 expression in the full cohort (*n* = 62).

### 2.10. Immunohistochemistry-Based Comparison of Extracellular Matrix in High and Low CEP68 Groups

**Figure 5:**
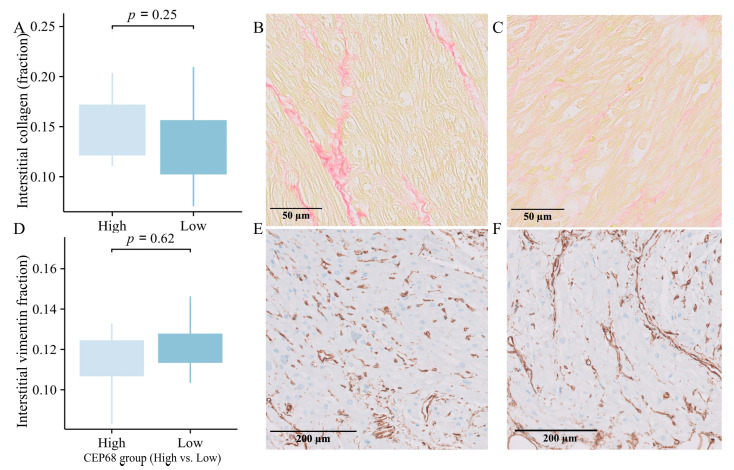
Immunohistology. (**A**) Interstitial collagen showed a non-significant increase in the high CEP68 groups in 22 no-AF patients, with error bars indicating the standard error of the mean. (**B**,**C**) Representative images of interstitial collagen in the high (left) and low (right) CEP68 groups. (**D**) The level of vimentin-positive interstitial area showed no significant difference between the high and low CEP68 expression groups in 22 no-AF patients. (**E**,**F**) Representative images of vimentin-positive interstitial cells.

For all 62 patients, *CEP68* expression were divided into high and low groups based on the median. The high *CEP68* group had a higher, though not statistically significant, level of interstitial collagen (16.50 ± 5.15% vs. 15.46 ± 4.69%, *p* = 0.41). For the 22 patients without AF, *CEP68* levels were similarly divided into high and low groups based on the median. Interstitial collagen showed no significant difference between the two groups (15.39 ± 4.22% vs. 13.28 ± 4.08%, *p* = 0.25) (Figure 5A–C). Additionally, there was no significant difference in the level of vimentin-positive interstitial area between the high and low *CEP68* expression groups, either in all 62 patients or specifically in the no-AF patients (11.71 ± 2.14% vs. 12.08 ± 1.25%, *p* = 0.62) (Figure 5D–F).

## 3. Discussion

In this study, we leveraged large-scale genomic and multi-omic datasets to identify primary cilia-related genes with potential causal roles in AF. Our analyses highlighted in particular *CEP68*, for which Mendelian randomization and colocalization suggested robust causality for AF, both through altered gene expression and through gene methylation. The role of CEP68 as a potential AF biomarker was subsequently supported by associations of CEP68 expression in LAA with AF and AF-related endophenotypes, within RNA sequencing data from prospective cohorts (the PREDICT-AF and MARK-AF studies). We further showed positive associations between CEP68 and cardiac remodeling biomarkers, such as THBS2, as well as significant upregulation of CEP68 in fibroblasts from AF patients. Thus, we propose that CEP68 may be associated with AF occurrence, with fibrosis potentially acting as a mediator in this process.

Primary cilia act as cellular antennas, responding to forces such as stretch within the myocardium. The membrane tension on the primary cilia influences all aspects of their mechanosensory functions, including the SHH signaling pathway [11]. Our GSEA enrichment analysis of *CEP68*-correlates indeed showed an enrichment for the SHH signaling pathway. Both our study and others have demonstrated that primary cilia are associated with fibrosis [3,4,12]. Furthermore, the knockdown of IFT88 which disrupts ciliogenesis/primary cilia formation, enhances TGF-β-induced collagen expression [4]. It has also been demonstrated that profibrotic molecules, such as TGF-β, inhibit ciliogenesis by negatively regulating the essential ciliogenesis gene IFT88 [13]. Notably, SHH and TGF-β signaling can intersect with mechanosensing pathways and have been linked to circadian-regulated metabolic states that influence fibrotic remodeling, providing a broader regulatory context for our enrichment findings [8].

CEP68 plays an important role in the formation and function of primary cilia [14,15]. CEP68 plays a crucial role in maintaining centrosome cohesion, which is essential for cell division, microtubule organization, and the function of primary cilia. We demonstrated the association between CEP68 and AF using GWAS data from eQTL level (Gene Expression) and mQTL level (DNA Methylation). Interestingly, we observed this causal relationship not only in LAA tissue but also in blood, suggesting that blood-based CEP68 may capture systemic biology relevant to AF liability and could serve as a circulating molecular feature that complements integrative prediction models—rather than acting as an independent standalone predictor—pending external validation [16]. Another possible reason is that changes in gene expression in the blood may reflect systemic biological changes, thereby influencing the progression of the disease. Taken together, our data support a conceptual framework in which CEP68—through centrosome/primary cilium regulation—interfaces with mechanosensing and SHH–TGF-β profibrotic signaling to promote extracellular matrix remodeling and thereby increase AF susceptibility, although the precise molecular intermediates and causal steps require further experimental elucidation.

In the clinical cohorts, although we observed a significant positive correlation between *CEP68* and LAVI as well as cardiac remodeling biomarkers, no significant difference in *CEP68* expression was found among different AF stages. Possible reasons for this include the relatively small sample size of sequenced patients and the difficulty in controlling for single variables in a complex clinical environment, such as age, which may obscure potential relationships. Additionally, the negative feedback systems could potentially mask the true association, even if a causal relationship between *CEP68* and AF exists.

Our study has several limitations. First, to comprehensively include all potential primary cilia-related genes, we incorporated all genes labeled as such in GeneCards without filtering. While some genes are associated with primary cilia, they also play roles in other cellular processes. Second, although we used clinical data to validate the potential causal relationship between CEP68 and AF, the clinical population may not effectively control for individual variables, and gene expression levels may reflect complex underlying biological processes. Cellular and animal studies are necessary for validating causality. Additionally, we did not conduct PCR studies; while RNA sequencing data can somewhat substitute for PCR, PCR remains the gold standard for gene expression measurement. Finally, although SMR has its own limitations such as pleiotropy, we performed extensive sensitivity analyses to assess the relationship between CEP68 and AF. In addition, circadian, metabolic, and lifestyle-related variables were not available in the current datasets and thus could not be incorporated into integrative models. Future studies combining multi-omics with longitudinal lifestyle and metabolic timing measures may further refine the translational relevance of CEP68.

Importantly, AF pathogenesis is likely governed by a complex, multicellular and multi-molecular interaction network [17]. Accordingly, we view CEP68 as one component of a broader, multifactorial regulatory network underlying AF, consistent with contemporary cardiovascular systems-biology models of complex traits. Collectively, our findings are consistent with a model in which genetically predicted CEP68 regulation may contribute to AF susceptibility, with fibrosis as a potential mediator.

## 4. Materials and Methodss

### 4.1. Study Design

This study followed the Strengthening the Reporting of Observational Studies in Epidemiology (STROBE) guidelines (Supplementary Table S1) [18]. The study design and the workflow for selecting genetic variants and analytical methods are summarized in Graphic Abstract. In summary, we:

1. Leveraged GWAS data for SMR and colocalization, to investigate the potential causal role of primary cilia genes in AF.
2. Analyzed single-cell data to explore the cellular distribution of potential target genes.
3. Investigated the mechanisms of potential target genes using RNA sequencing data from patients with and without AF.

### 4.2. Identify the Set of Primary Cilia-Related Genes

To identify genetic predispositions related to primary ciliary dysfunction, we compiled a list of 1794 known primary cilia-related genes from the GeneCards database (https://www.genecards.org/; accessed on 10 January 2024) and relevant literature [19]. Our gene list is provided in Supplementary Table S2. Due to the absence of specific genomic data solely focused on primary cilia, we combined information from the GeneCards database with published research to include as many primary cilia-related genes as possible.

### 4.3. Data Sources

To perform SMR and colocalization, we collected QTL GWAS data for molecular traits, including QTLs for transcript expression (eQTLs), for protein abundance (pQTLs) and for DNA methylation (mQTLs); these molecular trait QTL data were sourced from various datasets and are described in detail below.

For the generation of eQTL instruments, genetic variants located within 1000 kb of the coding sequences were identified using summary statistics from the eQTLGen Consortium (https://www.eqtlgen.org/cis-eqtls.html/; accessed on 10 January 2024) and the GTEx project. The eQTLGen Consortium provided data on 10,317 trait-associated SNPs from 31,684 individuals from whole-blood [20], while GTEx offered insights into genetic associations and gene expression across 49 tissues in 838 individuals [21]. We utilized data from eQTLGen for blood expression, and GTEx for LAA expression.

mQTL instruments for primary cilia-related genes in blood were identified using summary data from a meta-analysis of two cohorts comprising a total of 1980 individuals of European ancestry, with mQTLs defined based on significant associations between SNPs and DNA methylation levels within a 1 Mbp range of the genes and annotated to their respective genes using the latest gene annotation databases [22].

Blood-based *cis*-pQTL instruments were collected from the deCODE Health study, which assessed genetic associations for 4907 protein aptamers in 35,559 Icelanders using the SomaScan platform [23]. pQTLs for LAA tissue were obtained from 118 patients scheduled for coronary artery bypass surgery and without additional procedures, within the AFHRI-B study [24]. *Cis*-SNPs were defined as SNPs within 1 Mb from the gene encoding the protein.

Genetic associations for AF were extracted from the largest GWAS meta-analysis of 60,620 AF cases and 970,216 controls of European ancestry [25]. The GWAS summary data from FinnGen (40,594 cases and 168,000 controls) datasets were employed in the replication phase [26]. The details of all QTL and GWAS datasets for this study are presented in Supplementary Table S3.

### 4.4. Selecting Potential Genes from GWAS Data

We conducted our analysis based on previous SMR and colocalization studies [9]. We considered the core MR principles during study design: a strong instrument-exposure association, no confounding by the instrument, and the instrument affecting the outcome only through exposure [27].

For causal inference, we estimated effect sizes using SMR and derived odds ratios (OR) for primary cilia dysfunction from SNP effects on dysfunction and AF. The SMR analysis and heterogeneity in dependent instruments (HEIDI) test were performed using version 1.3.1 of the SMR software (https://yanglab.westlake.edu.cn/software/smr/#Download; accessed on 10 January 2024) [9]. To correct for genome-wide type I errors and keep the false discovery rate (FDR) below 0.05, we applied the Benjamini–Hochberg (BH) method for multiple testing corrections. Significant results were those with P_SMR below the threshold after BH correction for FDR < 0.05. We further verified these associations using the HEIDI test (P_HEIDI > 0.01) to determine if they were due to a shared causal variant rather than pleiotropy. The significance threshold after BH correction was determined based on the number of tests performed. Associations with FDR-corrected *p*-values <0.05 and P_HEIDI >0.01 were considered strong candidates for causality, and were then subjected to colocalization analysis.

A posterior probability for hypothesis 4 (PP.H4) greater than 0.80 was considered to provide strong evidence for colocalization, indicating a significant likelihood that the genetic variants associated with the traits of interest share a common causal variant. The analysis was conducted using Bayesian methods implemented in the R package Coloc (version 5.2.3), with default prior probabilities set at 1 × 10^−4^ for a causal variant being associated with each trait and 1 × 10^−5^ for a causal variant being shared between traits [28].

To evaluate the causal direction associated with SNPs, we utilized the Steiger method implemented in the TwoSampleMR R package (version 0.6.8), which compares the proportion of variance (R^2^) explained by the SNPs in the exposure and outcome datasets to infer the most likely causal direction. This method evaluates the direction of causality by comparing the variance explained by genetic variants in both the exposure and the outcome. A *p*-value threshold of 0.05 was established to determine statistical significance, allowing us to identify robust causal relationships between the genetic variants and the traits of interest.

### 4.5. Single-Cell RNA Sequencing Data Analysis

We obtained single-cell RNA sequencing data from the NCBI Gene Expression Omnibus database (Accession number GSE224959). This dataset comprises LAA tissue samples from five control individuals and seven patients with persistent AF who were undergoing cardiac surgery [29]. Single-cell RNA sequencing data were analyzed using Seurat (version 5.1.0) and SingleR (version 2.2.0) packages [30]. During quality control, cells with mitochondrial gene expression > 5% or fewer than 200 detected genes were removed [31]. The 1500 most variable genes were selected for Principal Component Analysis, followed by uniform manifold approximation and projection for dimensionality reduction [31]. Differential expression analysis compared selected genes between control and AF groups across cell types, with results visualized graphically. Our analysis focused on the cellular specificity of gene clustering as well as the distributional variances between the patients with persistent AF and the control group.

### 4.6. Data from the PREDICT-AF and MARK-AF Studies

RNA sequencing was performed on LAA samples from 62 patients, including 22 without a history of AF from the PREDICT-AF study and 21 with paroxysmal AF and 19 with persistent AF from the MARK-AF registry [32,33,34]. Paroxysmal, persistent, and longstanding persistent AF were defined according to the European Society of Cardiology guidelines [35]. However, for the current analysis, patients with persistent and longstanding persistent AF were grouped together as persistent AF [35]. The differential expression analysis compared three study groups: control, paroxysmal AF, and persistent AF, and was corrected for age and sex (R package Limma, version 3.56.2).

The PREDICT-AF study is a prospective cohort study and included 150 patients without AF who underwent cardiac surgery and LAA excision and were followed for two years [32]. Out of these 150 patients, LAA samples were used for RNA sequencing in 22 patients, 5 of whom developed AF during the follow-up. The MARK-AF study included patients with paroxysmal and persistent AF who underwent thoracoscopic ablation and LAA excision [34].

The correlation between potential target genes and clinical biomarkers—including C-reactive protein, NT-proBNP, CHA_2_DS_2_-VASc score, leukocyte count, age, LAVI, and LVEF—was assessed. In the 22 patients without AF from the PREDICT-AF study, three blood biomarkers associated with cardiac remodeling—Tenascin-C (TNC), Collagen VIII alpha 2 (COL8A2), and THBS2—were measured using ELISA [33]. Immunohistochemistry was performed to analyze interstitial collagen and vimentin-positive interstitial area in all 62 patients [34].

### 4.7. Gene Set Enrichment Analysis

To explore the biological pathways associated with selected genes in a network approach, we computed the correlation coefficients between the target gene and all other genes across the transcriptome, resulting in a set of correlation values for each gene. Subsequently, we ranked all genes based on their correlation coefficients, and pre-defined gene sets from the Molecular Signatures Database (MSigDB) (https://www.gsea-msigdb.org/gsea/msigdb; accessed on 30 January 2024) were used. Specifically, we utilized the *c2.all.v2022.1.Hs.symbols.gmt* dataset, which includes curated pathways and contains 6449 gene sets. Enrichment scores were calculated for each gene set, reflecting the extent of over-representation at the top or bottom of the ranked gene list. Statistical significance was assessed using permutation tests, and NES were computed to allow for comparison across gene sets. Gene sets with an FDR < 0.05 were considered significantly enriched.

### 4.8. Statistical Analysis

Quantitative data were compared using Mann–Whitney U or *t*-tests. Correlation analyses were conducted using Pearson’s or Spearman’s coefficients, depending on the data distribution. For comparisons involving multiple groups, One-way ANOVA was used when both normality (assessed via Shapiro–Wilk test) and homogeneity of variance (checked with Levene’s test) were satisfied. When normality was met but the homogeneity of variance was not, Welch’s ANOVA was applied. In cases where normality was not satisfied, the Kruskal–Wallis test was employed. Missing values were imputed using group means. As a sensitivity analysis, we repeated the analyses using complete-case data. Statistical significance was set at a *p*-value below 0.05. All statistical analyses were performed using R software (version 4.1.2 for Mac) and Python (version 3.12.0).

## Data Availability

The original contributions presented in this study are included in the article/Supplementary Material. Further inquiries can be directed to the corresponding author.

## Supplementary Materials

The following supporting information can be downloaded at: https://www.mdpi.com/article/10.3390/ijms27031498/s1.

## Abbreviations

The following abbreviations are used in this manuscript:

AF, atrial fibrillation; COL8A2, collagen type VIII alpha 2; ECM, extracellular matrix; eQTL, expression quantitative trait loci; FDR, false discovery rate; GSEA, gene set enrichment analysis; GWAS, genome-wide association studies; HEIDI, heterogeneity in dependent instruments; IFT88, intraflagellar transport protein 88; LAA, left atrial appendage; LAVI, left atrial volume index; LVEF, left ventricular ejection fraction; MR, Mendelian randomization; mQTL, methylation quantitative trait loci; NES, normalized enrichment score; NT-proBNP, N-terminal pro–B-type natriuretic peptide; pQTL, protein quantitative trait loci; PP.H4, posterior probability for hypothesis 4; QTL, quantitative trait locus/loci; SMR, summary-data-based Mendelian randomization; SNPs, single nucleotide polymorphisms; THBS2, thrombospondin-2; TNC, tenascin-C.
