# Supplementary material for: Atrial Fibrillation and Primary Cilia-Associated Genes: The Role of CEP68
Source: Int J Mol Sci. 2026 Feb 3;27(3):1498. doi: 10.3390/ijms27031498 (PMC12897887; doi:10.3390/ijms27031498)
Supplement: Supplementary file 1 [file ijms-27-01498-s001.zip › ijms-4119516-supplementary.pdf]

Supplementary Materials

Supplementary Table S1

STROBE-MR checklist table

Item No.	Section	Checklist item	Relevant text from manuscript
1	**TITLE and ABSTRACT**	Indicate Mendelian randomization (MR) as the study's design in the title and/or the abstract if that is a main purpose of the study	Detailed in the Title and Abstract sections
	**INTRODUCTION**		
2	**Background**	Explain the scientific background and rationale for the reported study. What is the exposure? Is a potential causal relationship between exposure and outcome plausible? Justify why MR is a helpful method to address the study question	Detailed in the Introduction section
3	**Objectives**	State specific objectives clearly, including pre-specified causal hypotheses (if any). State that MR is a method that, under specific assumptions, intends to estimate causal effects	Detailed in the Introduction section
	**METHODS**		
4	**Study design and data sources**	Present key elements of the study design early in the article. Consider including a table listing sources of data for all phases of the study. For each data source contributing to the analysis, describe the following:	
	a)	Setting: Describe the study design and the underlying population, if possible. Describe the setting, locations, and relevant dates, including periods of recruitment, exposure, follow-up, and data collection, when available.	Detailed in the Methods and Supplementary Table S3
	b)	Participants: Give the eligibility criteria, and the sources and methods of selection of participants. Report the sample size, and whether any power or sample size calculations were carried out prior to the main analysis	
	c)	Describe measurement, quality control and selection of genetic variants	Detailed in the Methods
	d)	For each exposure, outcome, and other relevant variables, describe methods of assessment and diagnostic criteria for diseases	

	e)	Provide details of ethics committee approval and participant informed consent, if relevant	Detailed in the Methods: Ethics and Supplementary Table 1
5	**Assumptions**	Explicitly state the three core IV assumptions for the main analysis (relevance, independence and exclusion restriction) as well assumptions for any additional or sensitivity analysis	Detailed in the Methods
6	**Statistical methods: main analysis**	Describe statistical methods and statistics used	Detailed in the Methods
	a)	Describe how quantitative variables were handled in the analyses (i.e., scale, units, model)	
	b)	Describe how genetic variants were handled in the analyses and, if applicable, how their weights were selected	
	c)	Describe the MR estimator (e.g. two-stage least squares, Wald ratio) and related statistics. Detail the included covariates and, in case of two-sample MR, whether the same covariate set was used for adjustment in the two samples	
	d)	Explain how missing data were addressed	
	e)	If applicable, indicate how multiple testing was addressed	
7	**Assessment of assumptions**	Describe any methods or prior knowledge used to assess the assumptions or justify their validity	Detailed in the Methods
8	**Sensitivity analyses and additional analyses**	Describe any sensitivity analyses or additional analyses performed (e.g. comparison of effect estimates from different approaches, independent replication, bias analytic techniques, validation of instruments, simulations)	Detailed in the Methods
9	**Software and pre-registration**		
	a)	Name statistical software and package(s), including version and settings used	Detailed in the Methods
	b)	State whether the study protocol and details were pre-registered (as well as when and where)	Detailed in the Methods
	**RESULTS**		
10	**Descriptive data**		

	a)	Report the numbers of individuals at each stage of included studies and reasons for exclusion. Consider use of a flow diagram	Detailed in the Results and Supplementary Table S3
	b)	Report summary statistics for phenotypic exposure(s), outcome(s), and other relevant variables (e.g. means, SDs, proportions)	
	c)	If the data sources include meta-analyses of previous studies, provide the assessments of heterogeneity across these studies	
	d)	For two-sample MR:<br>i. Provide justification of the similarity of the genetic variant-exposure associations between the exposure and outcome samples<br>ii. Provide information on the number of individuals who overlap between the exposure and outcome studies	
11	**Main results**		
	a)	Report the associations between genetic variant and exposure, and between genetic variant and outcome, preferably on an interpretable scale	Detailed in the Results, Supplementary tables and Supplementary figures sections.
	b)	Report MR estimates of the relationship between exposure and outcome, and the measures of uncertainty from the MR analysis, on an interpretable scale, such as odds ratio or relative risk per SD difference	
	c)	If relevant, consider translating estimates of relative risk into absolute risk for a meaningful time period	
	d)	Consider plots to visualize results (e.g. forest plot, scatterplot of associations between genetic variants and outcome versus between genetic variants and exposure)	
12	**Assessment of assumptions**		
	a)	Report the assessment of the validity of the assumptions	Detailed in the Results.
	b)	Report any additional statistics (e.g., assessments of heterogeneity across genetic variants, such as I^2, Q statistic or E-value)	
13	**Sensitivity analyses and additional analyses**		

	a)	Report any sensitivity analyses to assess the robustness of the main results to violations of the assumptions	Detailed in the Methods, Results, Supplementary methods and Supplementary tables
	b)	Report results from other sensitivity analyses or additional analyses	
	c)	Report any assessment of direction of causal relationship (e.g., bidirectional MR)	Detailed in the Discussion section
	d)	When relevant, report and compare with estimates from non-MR analyses	
	e)	Consider additional plots to visualize results (e.g., leave-one-out analyses)	Detailed in the Results
	**DISCUSSION**		
14	**Key results**	Summarize key results with reference to study objectives	Detailed in the Discussion section
15	**Limitations**	Discuss limitations of the study, taking into account the validity of the IV assumptions, other sources of potential bias, and imprecision. Discuss both direction and magnitude of any potential bias and any efforts to address them	
16	**Interpretation**		
	a)	Meaning: Give a cautious overall interpretation of results in the context of their limitations and in comparison, with other studies	
	b)	Mechanism: Discuss underlying biological mechanisms that could drive a potential causal relationship between the investigated exposure and the outcome, and whether the gene-environment equivalence assumption is reasonable. Use causal language carefully, clarifying that IV estimates may provide causal effects only under certain assumptions	
	c)	Clinical relevance: Discuss whether the results have clinical or public policy relevance, and to what extent they inform effect sizes of possible interventions	
17	**Generalizability**	Discuss the generalizability of the study results (a) to other populations, (b) across other exposure periods/timings, and (c) across other levels of exposure	
	**OTHER INFORMATION**		
18	**Funding**	Describe sources of funding and the role of funders in the present study	Detailed in the Abstract section

		and, if applicable, sources of funding for the databases and original study or studies on which the present study is based	
19	**Data and data sharing**	Provide the data used to perform all analyses or report where and how the data can be accessed, and reference these sources in the article. Provide the statistical code needed to reproduce the results in the article, or report whether the code is publicly accessible and if so, where	Detailed in the Data Availability
20	**Conflicts of Interest**	All authors should declare all potential conflicts of interest	Detailed in the Competing interests

Supplementary Table S2

primary cilia genes

V1	V2	V3	V4	V5	V6	V7	V8
CIMAP3	CEP290	INVS	CSPP1	PKHD1	RPGRIP1 L	CC2D2A	TMEM23 7
TCTN2	TMEM23 1	OFD1	EVC2	KIF3A	CEP164	MKS1	ARL13B
B9D1	PKD1L1	BBS1	GPR161	IFT140	KIAA058 6	B9D2	CEP78
PKD2L1	TCTN1	TULP3	IQCB1	IFT122	TMEM10 7	DCDC2	NPHP1
BBS4	SCNM1	TMEM67	OCRL	ALMS1	RRP7A	EVC	RAB8A
BBS2	FLCN	IFT88	SHH	PIBF1	IFT74	WDR19	RILPL1
BBIP1	LOC1268 63212	TMEM17	CCP110	IFT20	HDAC6	ENTR1	RPGR
NPHP3-ACAD11	TCTN3	ARL6	PKD1	EFCAB7	AURKA	SPAG17	TTLL3
ADCY3	TMEM21 6	PKD2	BBS5	NPHP4	AHI1	RPGRIP1	TMEM13 8
BBS7	IFT52	UMOD	VHL	DZIP1	BBS9	EXOC4	SDCCAG 8
TTC8	TTC21B	TRA-TGC7-1	IFT46	WDR35	BBS10	TXNDC1 5	EXOC3L2
RLIG1	LOC1019 28861	PCNT	TGFB1	PCM1	PLK1	CEP19	RAB10
MC4R	FUZ	CEP97	CTNNB1	BDNF-AS	SCAPER	DYNC2I1	DCTN1
DYNLT1	PRKCSH	TTLL6	KIF3B	CEP41	LCA5	PTCH1	CCDC13
DYNC2LI 1	SMAD5-AS1	GLI1	EGLN2	SEC63	C10orf90	RILPL2	RAB3IP
INPP5E	GLI3	PDE6D	EXOC5	POC1B	CEP162	DYNC2I2	RTTN
TUBA1B	ODF2	IFT43	SSTR3	CSNK1D	MCHR1	TRPV4	CSNK1E
ARFGEF1	CNTRL	ALG8	DHH	CFAP53	TAPT1	MXRA8	PPP1R35
ENSG000 00260092	TEK	LRRK2	PTK2B	RAB6A	ZDHHC2 4	SMO	AKT1
WNT5A	TUBB	TNFAIP8 L2-SCNM1	MYH10	TTBK2	NOL6	LUZP1	WNT3A
SSX2IP	TMX2-CTNND1	L1CAM-AS1	LINC0167 2	GSK3B	DVL2	CILK1	TOGARA M1

V1	V2	V3	V4	V5	V6	V7	V8
EGFR	PRDX1	RAB11A	ATAT1	CERNA3	TUBG1	CEP350	CALM2
PARVA	CALM3	STIL	MIR7- 3HG	CETN1	ERICH3	FFAR4	PDGFRA
CALM1	SPEN	CEP43	FAM161A	KIF7	PDGFRB	SMAD3	HDAC2
SMAD2	KIF11	CDK1	RUNX2	TUBB2A	CHMP4B	UBC	CBY1
UBR5	WWTR1	TPTEP2- CSNK1E	FAT4	NPHP3	DRD2	GLI2	TTN
SIRT2	DRD5	TIE1	DRD1	DRD4	MYO5A	BCAR1	DISC1
DRD3	PDGFA	SALL1	SMPD3	DAB2IP	CIP2A	HASPIN	NPFF
PKD1L2	RABL2B	PWAR1	APC	SIRT1	DIAPH1	INPP5B	EML1
LIMA1	TRAPPC9	DCAF1	KIF13B	ATOH1	TRP- AGG2-5	TRE- TTC3-1	IFT80
NIN	KIF24	CDK5	RAB7A	PDCD1	DDB1	VDAC1	TUB
SLC4A7	TRAPPC4	TRAPPC1 0	TRAPPC3	TRAPPC2 L	TRAPPC1	TRAPPC6 A	TULP2
KATNIP	RABL2A	TMEM10 2	LINC- ROR	GPR37L1	ALPK1	IQCE	LRGUK
TTLL7	GPR157	TTC23	IFT172	FBF1	KAAG1	ARL3	CEP83
DYNC2H 1	CEP89	NEK8	IFT57	SOX9	ILK	SLC6A6	LIMS1
NIM1K	UBL3	LGALS7	NUDT16L 1	TTN-AS1	HOXA@	HOXD@	MKKS
TRAF3IP 1	SCLT1	C2CD3	RHO	HTR6	CEP120	CPLANE1	DNAH11
CEP152	CNTROB	CNTLN	CEP20	CLDN1	ATXN10	RFX2	DNAH5
PALS1	PATJ	POC5	BBS12	EMSLR	KIAA075 3	LOC1053 71046	TBC1D7
TBC1D30	LOC1268 62260	NEK9	LEFTY2	ZIC3	EMX2	NEDD9	NODAL
VANGL2	DNAI1	ALX4	NKX2-2	SEPTIN4	SIX2	IFT27	WDPCP
CFC1	DCHS1	EXOC1	FOXJ1	IFT56	KIF17	VAX1	DAND5
SHROOM 3	ANKS3	KIF19	ALX3	HIVEP3	CC2D2B	IFT22	TMEM21 8
ARL13A	USH1G	LZTFL1	ENPP1	GAS8	RAB23	AIPL1	TMSB4X
CROCC	EVI5L	KIF14	ASAP1	MAPK15	YIF1B	TTC21B- AS1	LOC1268 60772

V1	V2	V3	V4	V5	V6	V7	V8
IGF1R	SPTAN1	GRK2	CSPG4	OPTN	ARL2	ADAMTS 9	DCTN2
RAB8B	CEP170	EVI5	PLA2G3	CEP131	PRKACA	GSN	PLK4
ARRB1	CDK10	ACTR3	IFT81	PTPN23	TBC1D1	CEP250	GLIS2
RAB14	RAB1A	TTLL5	SGSM3	TBC1D24	USP6NL	ARL2BP	CEP104
RAB17	TBC1D20	AVIL	TBC1D14	TBC1D15	TBC1D5	CIBAR1	RAB15
RABGAP 1	TBC1D10 A	TBC1D10 C	TBC1D2	TBC1D22 A	TBC1D2B	TBC1D21	TBC1D22 B
TCHP	ZFYVE19	TBC1D8	TBC1D13	TBC1D16	TBC1D17	TBC1D10 B	TBC1D9B
TBC1D19	TBC1D3	TBC1D7-LOC1001 30357	MTOR	NEK2	CUL3	ARRB2	HTT
PCSK2	NEK1	XPNPEP3	CETN2	HAP1	JADE1	PCARE	TBC1D31
CEP295	NFE2L2	TSC2	ITGB1	NOG	NPHS1	KCNH1	ORC1
MAP1B	MAPRE1	CDKL5	NDE1	RANBP1	TCP1	CCT4	CENPJ
DACH1	KCTD17	MAP4	RFX1	KIZ	NINL	RAB34	CEP192
PROK1	DZIP1L	MPHOSP H9	TPRA1	TTC21A	ODAD1	CEP128	TAS2R43
TBC1D32	KRTCAP 3	TERC	ADAMTS 9-AS2	ADAMTS 9-AS1	TRD-GTC9-1	LOC1268 06961	LOC1268 62216
CDKN1B	CSNK1A1	HSPA9	TSC1	YWHAB	DPYSL2	MECP2	PROM1
CLTC	DYNC1H 1	CCT5	HERC2	HSPA1L	KRT17	LAMA5	MIB1
CENPF	HNF1B	PLEC	RAN	AKAP9	CCT2	FABP4	HSPA4
PRPF31	SSB	TRIP11	CAPZA1	CCDC88 A	CCT3	GPC5	HAVCR1
KHSRP	LGALS3B P	NEB	PDE4DIP	RPL27A	SYNE1	CAPRIN1	DYNC1LI 2
FRAS1	ACTR1A	ATAD3B	CLTRN	HECW1	PJA2	POC1A	POF1B
SEPTIN7	SPRR3	CEP112	SASS6	TBCCD1	ODF2L	TTC17	CEP44
FOXK1	NEK5	CATIP	CCDC66	CIBAR2	CEP95	SFTA3	MIR21
MIR146B	TRIM59-IFT80	RAF1	FLT3	EZH2	EP300	PTEN	CBL
GJA1	NPM1	PCNA	PRKAR1 A	PRKDC	VIM	AIFM1	CAD

V1	V2	V3	V4	V5	V6	V7	V8
HSPA8	KCNQ1	P4HB	PCSK1	SLC2A2	SNAP25	SQSTM1	YWHAE
ACACA	ACE2	BLM	BMP1	CACNA1S	GFAP	HSPA5	HSPD1
KEAP1	RPL11	TUBA1A	YWHAG	CYLD	ENO1	KRT1	MYH7
MYH9	PC	POLH	SMC1A	SMC3	TUBA4A	TUBB4A	XPO1
YAP1	ATP5F1A	DLAT	FER	HUWE1	LBR	LIFR	PAFAH1B1
PKLR	PKN2	PLOD3	POU5F1	SLC25A12	USP8	ADAR	ANO1
CANX	CBLB	CDC6	CDH23	GARS1	ISCU	THBS1	TK1
TKT	AGRN	CANT1	CLIP1	DDX6	HTRA1	LCN2	LIMK2
MKI67	NONO	NUP214	REV3L	RUVBL1	RUVBL2	SF3B1	SLC25A3
XRCC5	CARS1	CASQ1	CDT1	CUL1	HSPA2	KIF2A	PRDX5
RACK1	REST	RPS20	SLC25A11	SUFU	TUBB2B	TUBB4B	UNC119
VASP	ADGRV1	ATG16L1	ATP6V0D1	CYP2J2	DOK2	EHHADH	ERC1
FABP7	FANCI	FREM1	GABARAP	HNRNPD	INHA	MACF1	MATR3
ORC4	PSMA1	PSMA2	PTPA	RP2	RPS12	SH3KBP1	SULT1A1
TRIM37	ARF4	CAP1	CCT7	CDC14A	CDK20	CTNNA3	DYNC1I2
ELAVL1	ESPL1	ETS2	GABARAPL2	KIFAP3	MAP1LC3B	NUP85	PCBP2
PLS1	PTMA	RPS16	RPS23	RTEL1	RUNX1T1	SEPTIN9	SLC25A6
SPRY1	ARID5B	ATP6V1D	BAG2	CAPZB	COL27A1	EHD1	EXOC6
IRS4	ITSN1	MYL6	PIEZO1	PRPF4B	RFX3	RIOK1	RPL22
SNX10	SST	TESK1	VIL1	ADGRD1	ALDOC	AMOT	ANKS6
ATP5PB	CAPZA2	DSPP	EN1	FLVCR1	FYCO1	MYO10	ORC6
PPP1R2	PSMD11	ZNF423	BCO1	BICD1	CKAP5	CYP3A43	DCTN4
DCTN5	DLGAP4	DTX1	DYNC1LI1	HMCN1	HOXA9	HSPA1B	HYDIN
ILF2	ILF3	PRPF19	SEPTIN2	VSX1	ZMYND10	ACTR1B	ARMC9
CCDC8	CGNL1	DCTN6	DDX19B	DENND3	DLEC1	DYNLRB1	EAF2

V1	V2	V3	V4	V5	V6	V7	V8
ESCO1	FOXK2	H1-1	MAPRE3	NAP1L1	PPFIA1	PPP1R13 B	RAB11FI P3
SNAPIN	TCF20	ADAMTS 20	APEX2	ARHGAP 19	CCAR2	CEP85L	CLUAP1
FAT2	FBXL13	GOLGB1	HP1BP3	HYCC1	NUP188	PGAM5	ROPN1L
STX18	ZNFX1	ACTL7B	ATP9B	BCL9L	CEP85	DCTN3	EML5
ETV3	GPATCH 8	IFT25	IGDCC4	KCTD10	KLHL18	LRRCC1	MAP3K21
RAB43	ROMO1	THAP2	TRIM10	VAT1	FKBP15	KCTD6	KLHL21
SLTM	TUBGCP 5	VPS13C	ZNF333	BLTP1	FAM161B	KLHDC8 A	MIS18BP 1
BEND7	CCDC14	CCNQ	FAM149B 1	MICAL3	MT-ND5	ZNF251	CCDC77
CNST	KCTD11	LRRIQ1	MT-ND4	PODNL1	WDSUB1	ZNF287	ANKRD3 1
DZANK1	SAXO4	TAS2R4	TAS2R46	GAB4	PYDC1	TEDC1	CFAP157
TEDC2	NPIPA1	ANKRD1 3C-DT	CLPSL2	MOSMO	TRA	TRB	MIR34B
MIR34C	MEG8	NPHP3-AS1	SNORD15 A	LINC0115 2	KIZ-AS1	TRC-GCA24-1	TRN-GTT2-1
LOC1073 03340	LOC1268 06174	LOC1268 06306	AKT3	FGFR3	KIT	NTRK2	KRAS
MYC	SLC2A1	CAMK2A	DDR2	HSP90AA 1	PIK3CA	TNF	XIAP
APP	CA2	EPAS1	G6PD	GAPDH	MAP3K7	MMP14	PARP1
PRKCA	TFRC	ACTB	AHCY	BDNF	BUB1B	CHUK	EIF2AK3
GSTP1	ITGB4	JUN	LMNA	LPL	MAPK8	MMP1	PIK3R2
PKM	RHEB	USP7	VEGFA	ACTN1	ALB	CACNA1 H	CAMK2D
CD44	CHRM3	EEF2	FASN	FGG	FLNA	GSK3A	HIF1A
HSP90AB 1	IGF1	IMPDH2	NF1	PIP5K1C	PRKACB	PRKD1	ROR2
STK4	TOP1	TUBB3	UBA1	USP9X	ACLY	ACTA1	ACTA2
ACTG1	ANXA1	CAMK2G	CPT1A	CTPS1	HNRNPA 1	MAPK7	MCM4
NR1H4	PARK7	PGD	PLA2G6	PTK2	RPA1	RPL5	TPI1

V1	V2	V3	V4	V5	V6	V7	V8
TTK	UBE2I	YWHAZ	ACAN	ACTN4	ALDH3A1	ALDH3A2	AP2M1
APOB	BIRC5	CACNB2	CFL1	DDR1	DLST	EIF4A1	ERCC6
EZR	FEN1	GHR	HCFC1	HNRNPA2B1	HSD17B10	KRT8	LYZ
MAP4K2	OGDH	PEBP1	PFN1	PML	PRKAA1	RFC1	RPSA
SLC22A5	SLC25A13	SRPK1	TGIF1	TMPO	YWHAH	ATP2A3	CDC20
COL6A3	DYRK1B	EIF2S3	FBXW7	GNB1	GNPAT	HYOU1	LTF
MCM3	MCM5	NAGA	OSMR	PHB1	PPP1CC	PRKAR2A	SFN
SP1	STUB1	XYLT2	ZYX	AARS1	ABCB7	ACTC1	AGPAT2
AHSG	ANK1	ATP5F1B	ATP6AP2	CRBN	EEF1A1	EMD	FBXW11
FOXG1	FUS	GDI1	LTBP1	MSR1	MTHFD1	NDUFS1	NEFH
NME2	NRCAM	NSF	NUP98	PALB2	PDK3	PFKFB3	PFKL
PLOD1	PPARA	PPP2CB	PSMA6	PSMA7	PSMB1	PSMD4	PXDN
RAB5A	RBBP4	RPL15	RPL35	RTN4R	SKP2	SLC1A4	STK24
STK3	TDP2	TRIM28	TRIO	TUBA8	TUFM	UBE2D1	USP1
XRCC6	ACTG2	ACTR2	ANP32A	CCNB2	CDC37	CIITA	CLN3
COPB2	CORO1A	COX10	CUL2	CUL4A	DOK1	ERLIN2	FOXP2
GIPC1	H2AX	HSPA6	IQGAP1	KCNH7	KMT5A	KPNB1	KRT19
MAFB	MYO6	MYO7A	MYOF	NID1	NLRP1	NOP56	NUDC
PAPOLA	PARP4	PDIA3	PNPLA6	PPM1B	PPP6C	PRKAR2B	PSMA4
PSMB5	PSMB7	PSMC1	PSMC3	PSMD14	QPCT	RBX1	RPL10
RPL18	RPL21	RPN1	RPS24	RPS27	RPS3	SF3B4	SFPQ
SLC25A5	TIMP3	TRAP1	TRIM21	TRIM32	UGT8	VDAC3	ABCA12
AGTPBP1	ANO6	AP2B1	BCL11B	BMP15	CACNB1	CALU	CEP57
CNOT3	COPA	CUL5	DGKD	DHX9	DST	DYNLL1	EEA1
EEF1B2	EFTUD2	HAT1	KDM2B	KLC1	LIPG	LRPPRC	MAK
MARK1	MDC1	MFN1	MGAM	MRPS16	MRPS22	PALLD	PASK
PCBP1	PFKFB2	PGM2	PHB2	PSMD12	PSMD3	PTBP1	RBBP7
RFC4	RPL27	RPL3	RPL35A	RPL8	RPS14	RPS15A	RPS17

V1	V2	V3	V4	V5	V6	V7	V8
RPS27A	SMC4	SNRNP200	SRSF1	STK38L	TACC3	TBC1D4	TLN1
TOPORS	USH1C	VPS35	ABCA2	ACTL6A	AFG2A	AKAP1	AP3B2
APC2	ARCN1	ARF3	ATAD3A	BMAL1	CADPS	CAMLG	CBR3
CHM	CLASP1	CLDN2	COPB1	CPSF1	CSE1L	CST6	DHX15
DHX30	DNAJB6	DSG4	EEF1D	EXOSC2	GNGT1	GRAP	HACE1
HMGN1	HNRNPC	HNRNPF	HNRNPH1	HNRNPR	HOMER3	HSPE1	IGF2BP3
IGSF1	JUNB	KTN1	MAGED1	MSRA	NCAPD3	NCAPG	NEK7
NME3	ONECUT1	PABPC4	PARD3	PIGR	PLS3	PRPF6	PSMC4
RAB3A	RABGGTA	RBCK1	RBL1	RBM10	RNF31	RPL10A	RPL7
RPS13	RPS9	SEC13	SETBP1	SF3B2	SIAH2	SNAP29	SNRPE
SRSF2	SSBP1	TNPO1	TRAF5	TUBGCP6	UBE2E1	ULK2	UPF1
WWP1	ANAPC7	ANKRD26	ANOS1	ARHGAP35	ARHGEF3	BCLAF1	CCT6A
CDC5L	CDK5RAP2	CEP55	CEP63	CERT1	CLSPN	CNGB3	COPE
COPG1	CYFIP1	DDX17	DMBT1	DSE	ENAM	EPN2	FOXD3
H2AZ1	HNRNPL	HYAL4	IGF2BP1	IMMT	JPH1	KCTD1	KIRREL1
MAPKBP1	MCPH1	MED14	MEOX1	MEST	MFAP4	MTDH	MTPAP
MYBBP1A	NEK4	NME7	NSMAF	OMG	PPP2R3C	PSMD13	RAB35
RAB5B	RAB5C	RCN2	REG1A	RFC3	RPL12	RPL23A	RPL24
RPL34	RPLP1	RPLP2	RPS2	RPS5	SETMAR	SH3PXD2A	SLC39A10
SLPI	SMC2	SMCHD1	SON	SPAG9	TIMELESS	TRAK2	TUBGCP4
UQCRH	YBX3	A2ML1	ADAMTS6	ARHGAP6	ATP5F1C	ATP5PF	CEP135
CORO1C	CPSF4	CSDE1	DBN1	DLG5	DYRK3	FARP2	FOXP4
GDI2	GPBAR1	GRPEL1	HNRNPM	IQGAP3	LARP1	LCN1	MPP1
MRPS25	MYL12A	NKAP	PSMC6	PSMD6	QSOX1	RBKS	RBM17

V1	V2	V3	V4	V5	V6	V7	V8
RBM4	RFC5	SGMS1	SHANK1	SLC47A1	STRAP	SYNPO	TIMM50
TNFRSF1 9	TTC12	TUBGCP 2	UBR4	YBX2	ZNF462	ABHD4	AKAP3
ANKFY1	ANP32B	ATPAF2	BAG6	BZW2	CCDC88C	CCL26	CDKL1
CDKL3	CELSR3	CETN3	CKAP2L	CLNS1A	CPSF6	DCAF7	DDX21
DDX50	DNAJA2	EIF5A2	ELOB	EXOSC7	HNRNPA 3	HYLS1	INTS11
INTU	KCTD7	KIF15	KLHL20	MPRIP	MTREX	NACA	NOP2
PATZ1	PDIA6	PDP2	PHC2	PIP	RBM39	RCAN2	RHOBTB 3
RNF41	RPS4X	SEC61B	SF3B3	SHARPIN	SLC47A2	SMG6	SNRPD1
SNRPD2	STRN3	SYNCRIP	TECTA	TENM1	TGIF2	TTBK1	TUBA3E
TUBGCP 3	TXNL1	UGT2A3	USP21	VPS18	ZC3HAV 1	ABCA13	ADGRB1
AKAP10	CDKL2	CEP72	CHML	CKAP2	COL5A3	CPA5	CRCP
DDX43	DERA	DOCK5	DYNLL2	E2F8	EBNA1B P2	EIF4ENIF 1	EPB41L5
ERLEC1	EXOC6B	FRMPD4	GLG1	GLIS3	GOLGA5	GRSF1	H2AC20
HAUS6	HECTD1	HNRNPU L1	IPO5	JCHAIN	KCMF1	KCTD15	KDM1B
KLHL1	KLHL13	KLHL22	KLHL40	LANCL1	MRPL23	MRPS18B	MRPS27
MYEF2	NEDD1	PIWIL4	RAB3D	RPL32	RPS18	RPS21	RPS25
RRP1B	SAV1	SCIN	SEPHS2	SF3A2	SH2D3C	SLC25A1 8	SLC30A7
SLC5A10	SNTB2	SRRM2	TNFAIP2	UACA	WHRN	YTHDF2	ZMYM4
ZNHIT6	ZRANB2	ACAD11	ACTR10	AKAP8L	ATG2B	BPIFA1	CALML5
CAPN15	CEP68	CEP70	CKAP4	CNN3	CSMD3	CSN1S1	DDRGK1
DEGS2	DNAAF2	DNAH1	EMSY	H2AZ2	HNRNPA 0	KCTD3	KIAA121 7
MAGEA1 0	MPP4	MRPS17	MRPS34	MTCL1	MYL12B	NASP	OTUD4
OXSM	PLEKHG 1	PNMA2	PPM1H	PSG6	RAB3B	RAB3C	RFX4
RPRD2	RUSC1	SCGB2A2	SHKBP1	SKIC8	SLAIN2	SMG7	SNRPD3
SPA17	TENT4B	TUBA3C	U2SURP	UPK1A	VPS52	ZBTB32	ACTBL2

V1	V2	V3	V4	V5	V6	V7	V8
AHCTF1	ALDH16 A1	ALLC	BTBD2	CHCHD4	CPNE9	CSN2	CTTNBP2 NL
ELMOD2	GCN1	GREB1L	HAUS1	HAUS8	HOOK2	HRNR	IGHG1
KCTD12	KCTD13	MIPOL1	MRPS26	MTERF1	NLRP14	NUDCD3	PIH1D1
RAB12	RCOR3	RNF112	RPAP3	RPL36A	SERBP1	SF3B5	SIDT1
SPECC1	SRP9	TUBAL3	XRN1	ABTB2	AGBL4	CABYR	CCDC136
CEP76	DUSP26	ECPAS	FBXO22	FBXO33	H2AC25	H2AC6	H2BC5
HAUS3	HAUS4	HAUS7	HELB	KLHL29	MZT2B	PCDHGC 5	PRPSAP2
RABIF	SPATA2	SSNA1	STRIP2	SYNRG	TAF7L	TCP11L2	TRIM56
TSPYL4	TTC33	UEVLD	WDR47	DYNC2I2	ZNF350	ASXL3	C2CD6
C7orf50	CAMSAP 3	FAM83G	H2AC1	HAUS5	MAP7D3	MTCL2	MZT2A
PARPBP	SH3BGR L2	SLF1	SPICE1	WDR75	ZGRF1	ACTRT1	ALPK2
ARHGAP 23	CCDC138	CCDC92	CENPV	FAM186B	FAM193A	H2AC12	H2AC13
H2AC7	H2AJ	H2BC12	HAUS2	IGKC	IQCH	KCTD4	KLHDC1 0
KLHL30	L3HYPD H	LACRT	LCA5L	LGSN	LRRC37B	LRRC45	MINDY2
PRR12	PRR4	RAP1GA P2	ROPN1	TBC1D12	TTC28	TTLL11	ZNF665
CCDC61	COPRS	FAM83C	H2AC14	KCTD18	KCTD21	POTEE	RGPD8
RTP3	SBSN	TTC16	USPL1	ZNF670	AGAP4	BTBD6	C21orf62
CCDC15	CCSAP	CMTM1	H2AC21	H2AC8	IGHA1	LRRC42	SLC35G1
STARD9	ZFP14	ABTB3	C21orf58	FAM217B	NBPF15	OR7G1	RBMXL1
RGPD3	SYT15	TPGS1	ANXA2R	GPATCH 11	NBPF1	NBPF11	OR4A16
POTEF	AGAP5	SPMIP2	TMEM86 A	CIMIP2A	NBPF12	TMEM20 0C	NBPF10
RGPD6	GRAPL	NBPF14	NBPF9	PVT1	CASC2	HEPN1	NBPF8
H2AC19	IGLC2	ZNF487	POTEKP	AGAP7P	MIR22	DEFB130 A	LINC0026 9
MIR141	MIR433	RPLP0P6	LINC0158 7	MIR193A	MIR200A	NPSR1- AS1	SNORD3 A

V1	V2	V3	V4	V5	V6	V7	V8
IGLV2-14	MIR1180	HOXB-AS1	SNORA59B	RAB4B-EGLN2	SNORD3B-1	EPN2-AS1	SNORD3C
LINC02166	PKD1P6	SNORD3B-2	SNORD3D	EPN2-IT1	LINC03009	AGAP14P	LINC02094
GRAPL-AS1	TRG-CCC3-1	TRW-CCA2-1	LOC388436	LOC102724624	LOC126806252	LOC126807151	LOC121852925
LOC121852926	LOC125177436	LOC125177437	LOC125177438	LOC125177439	LOC126862517	CCDC22	CCDC28B
HSPB11	KIF3C	MDM1	MOK	PROSER3	RAB29	RP1	RP1L1
SPATA7	TCTEX1D2	TMEM80	TTC26	TTC30A	TTC30B	TULP1	ULK4
WDR34	WDR60	WRAP73					

Supplementary Table S3

Information of QTL and GWAS datasets.

Type of dataset	Data subtype	Resource	Sample size	Reference
**QTL**	**cis-eQTL blood**	eQTLGen Consortium	31,684	Võsa U, et al. Nat Genet. 2021. PMID: 34475573. Large-scale cis- and trans-eQTL analyses identify thousands of genetic loci and polygenic scores that regulate blood gene expression.
	**cis-eQTL LAA**	The GTEx Consortium	838	The GTEx Consortium. Science.2020. PMID: 32913098.The GTEx Consortium atlas of genetic regulatory effects across human tissues.
	**cis-mQTL**	McRae et al. mQTL summary data	1980	McRae AF, et al. Sci Rep. 2018. PMID: 30514905. Identification of 55,000 Replicated DNA Methylation QTL.
	**cis-pQTL**	deCODE	35,559	Ferkingstad E, et al. Nat Genet. 2021. PMID: 37794188. Large-scale integration of the plasma proteome with genetics and disease.
		Assum, I, et al. pQTL summary data	118	Assum, I, et al. Nat Commun. 2022. PMID: 35064145. Tissue-specific multi-omics analysis of atrial fibrillation.
**GWAS summary**	**Atrial Fibrillation**	GWAS meta	case: 60,620, control: 970,216	Nielsen, J. B, et al. Nat Genet. 2018. PMID: 30061737. Biobank-driven genomic discovery yields new insight into atrial fibrillation biology
	**Atrial Fibrillation**	FinnGen	case: 40,594, control: 168,000	Kurki MI, et al. Nature. 2023. PMID: 36653562. FinnGen provides genetic insights from a well-phenotyped isolated population.

Figure S1

A

Gene	probeID	Instrumental.variant	QTL.size.effect	β.coefficient	OR (95% CI)	P_smr	FDR	Colocalization
AGPAT2	ENSG00000169692	rs12002299	-0.21	-0.18	0.83 (0.75 - 0.93)	9.88e-04	3.83e-02	1.95e-03
AKT3	ENSG00000117020	rs67027895	0.12	0.26	1.30 (1.13 - 1.49)	2.24e-04	1.08e-02	5.67e-01
ALB	ENSG00000163631	rs150643605	-0.11	-0.42	0.66 (0.53 - 0.81)	1.32e-04	7.23e-03	9.75e-01
ARHGAP19	ENSG00000213390	rs56189004	-0.43	0.05	1.05 (1.02 - 1.09)	1.44e-03	4.79e-02	7.69e-02
ARRB2	ENSG00000141480	rs55682338	0.48	-0.06	0.94 (0.91 - 0.97)	4.26e-04	1.90e-02	8.13e-01
CCDC136	ENSG00000128596	rs2270593	-0.29	0.14	1.15 (1.07 - 1.24)	8.92e-05	5.36e-03	7.78e-01
CCDC88C	ENSG00000015133	rs1285834	-0.10	-0.24	0.78 (0.68 - 0.91)	1.14e-03	4.09e-02	7.53e-01
CCDC92	ENSG00000119242	rs3768	0.28	-0.15	0.86 (0.81 - 0.92)	3.30e-06	3.60e-04	7.33e-01
CEP68	ENSG00000011523	rs74181299	-0.30	0.23	1.25 (1.18 - 1.33)	1.50e-12	1.81e-09	9.50e-01
CEP85L	ENSG00000111860	rs9489520	-0.23	0.11	1.11 (1.04 - 1.19)	1.09e-03	4.09e-02	2.70e-14
CLN3	ENSG00000188603	rs151228	0.20	-0.11	0.90 (0.84 - 0.96)	1.27e-03	4.37e-02	1.34e-01
FAM149B1	ENSG00000138286	rs3793663	0.41	-0.14	0.87 (0.81 - 0.93)	3.06e-05	2.30e-03	1.49e-19
HNRNPF	ENSG00000169813	rs2393949	-0.19	-0.15	0.86 (0.80 - 0.93)	7.69e-05	4.87e-03	9.44e-01
ILF2	ENSG00000143621	rs10908848	0.09	0.29	1.34 (1.14 - 1.56)	2.87e-04	1.33e-02	1.31e-04
MRPS16	ENSG00000182180	rs7100670	0.36	-0.15	0.86 (0.80 - 0.92)	3.38e-05	2.39e-03	1.59e-19
NACA	ENSG00000196531	rs4788	0.14	0.37	1.45 (1.27 - 1.67)	7.00e-08	1.12e-05	9.73e-01
PFKFB2	ENSG00000123836	rs1564879	0.07	-0.38	0.68 (0.56 - 0.84)	2.16e-04	1.08e-02	9.81e-01
PKD1	ENSG00000008710	rs13333747	-0.14	0.23	1.26 (1.10 - 1.46)	1.16e-03	4.09e-02	7.28e-03
PTK2	ENSG00000169398	rs10088133	-0.16	-0.23	0.79 (0.73 - 0.86)	7.47e-08	1.12e-05	7.25e-01
QPCT	ENSG00000115828	rs7593932	0.16	0.19	1.21 (1.11 - 1.32)	2.31e-05	1.93e-03	8.68e-01
RAB1A	ENSG00000138069	rs6546125	-0.06	0.78	2.18 (1.47 - 3.22)	9.70e-05	5.55e-03	6.73e-01
RAB29	ENSG00000117280	rs7522056	-0.34	-0.08	0.92 (0.88 - 0.96)	6.97e-05	4.65e-03	2.41e-01
REST	ENSG00000084093	rs13353552	0.06	-0.53	0.59 (0.44 - 0.78)	1.93e-04	1.01e-02	8.68e-01
RIOK1	ENSG00000124784	rs12212766	-0.23	-0.10	0.91 (0.86 - 0.96)	8.49e-04	3.40e-02	6.05e-01
RPS2	ENSG00000140988	rs6366	0.12	-0.40	0.67 (0.57 - 0.79)	2.23e-06	2.98e-04	9.44e-01
SF3B1	ENSG00000115524	rs3980095	0.13	0.26	1.30 (1.16 - 1.45)	3.76e-06	3.77e-04	8.29e-01
TTN	ENSG00000155657	rs13034990	-0.07	-1.24	0.29 (0.19 - 0.45)	5.65e-08	1.12e-05	6.55e-01

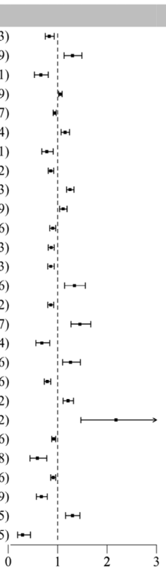

B

Gene	probeID	Instrumental.variant	QTL.size.effect	β.coefficient	OR (95% CI)	P_smr	FDR	Colocalization
CEP68	ENSG00000011523	rs74181299	-0.59	0.11	1.12 (1.08 - 1.16)	3.78e-11	6.18e-09	8.64e-01
PCM1	ENSG00000078674	rs574774	0.35	0.24	1.27 (1.12 - 1.43)	1.64e-04	7.64e-03	7.13e-06
CCDC92	ENSG00000119242	rs3768	0.41	-0.10	0.90 (0.86 - 0.95)	3.65e-05	2.67e-03	8.95e-01
PFKFB2	ENSG00000123836	rs1564879	0.42	0.06	1.07 (1.03 - 1.10)	1.16e-04	6.32e-03	9.32e-01
KDM1B	ENSG00000165097	rs34969716	0.43	0.16	1.18 (1.12 - 1.24)	2.86e-11	6.18e-09	1.00e+00
MRPS16	ENSG00000182180	rs7915521	0.18	-0.31	0.74 (0.62 - 0.88)	5.36e-04	1.95e-02	2.96e-07

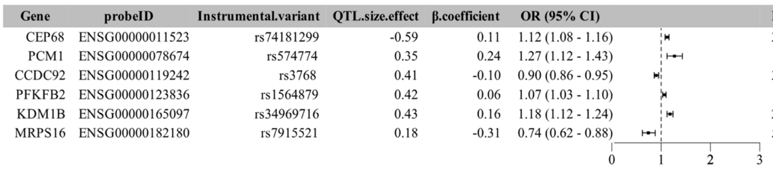

Fig. S1. Mendelian randomization results for the association between the expression of primary cilia genes and AF risk. (A) Blood sets and (B) Left Atrial Appendage (LAA) tissue sets. The figure represents the effect size (β) of a variant on mRNA expressions, where β > 0 indicates a positive association and β < 0 indicates a negative association. Odds ratios were calculated based on the expectation of the causal estimate (β coefficient). 'Colocalization' refers to the probability (PP.H4) of colocalization between eQTLs and AF outcomes.

Figure S2

Gene	probeID	Instrumental.variant	QTL.size.effect	β.coefficient	OR (95% CI)	P_smr	FDR	Colocalization
SDCCAG8	cg27259271	rs2994320	-0.24	-0.14	0.87 (0.80 - 0.94)	5.05e-04	2.62e-02	8.05e-01
SMG6;SRR	cg27564939	rs2209073	-0.48	-0.07	0.93 (0.91 - 0.96)	4.73e-06	8.69e-04	8.13e-01
PRKAR2B	cg26104690	rs2251838	-0.20	-0.15	0.86 (0.79 - 0.93)	4.36e-04	2.37e-02	8.35e-01
KIF7	cg18568647	rs4932232	-0.20	-0.13	0.87 (0.81 - 0.95)	8.45e-04	3.75e-02	8.36e-01
PRKAR2B	cg10691109	rs2536501	-0.30	-0.10	0.90 (0.86 - 0.95)	1.07e-04	7.41e-03	8.37e-01
CCDC92	cg11336643	rs1882491	-0.38	0.09	1.10 (1.06 - 1.15)	6.26e-06	1.04e-03	8.43e-01
PSMB7	cg01743846	rs2236386	-0.20	0.14	1.15 (1.07 - 1.24)	2.54e-04	1.56e-02	8.80e-01
RFX4	cg19965424	rs10778507	0.42	-0.07	0.93 (0.90 - 0.96)	6.32e-05	4.67e-03	8.80e-01
SDCCAG8	cg16104450	rs2994329	-0.38	-0.10	0.91 (0.87 - 0.95)	5.37e-05	4.15e-03	9.02e-01
ZNF487	cg27438128	rs1126709	0.33	0.08	1.08 (1.04 - 1.13)	2.54e-04	1.56e-02	9.12e-01
ZNF487	cg00588614	rs6593513	0.25	0.10	1.11 (1.05 - 1.18)	4.84e-04	2.58e-02	9.17e-01
CUL4A	cg14896830	rs9549696	0.29	-0.15	0.86 (0.80 - 0.92)	1.28e-05	1.51e-03	9.22e-01
TTN;MIR548N	cg09915519	rs744426	-0.36	-0.26	0.77 (0.71 - 0.84)	7.41e-10	7.90e-07	9.31e-01
MIR548N;TTN	cg10087519	rs12998857	-0.23	-0.36	0.69 (0.60 - 0.81)	2.36e-06	5.79e-04	9.35e-01
RFX4	cg15890332	rs9804754	0.92	-0.03	0.97 (0.95 - 0.98)	9.60e-06	1.31e-03	9.58e-01
SNHG9;SNORA64;SNORA78;RPS2	cg02871659	rs758335	-0.89	0.05	1.05 (1.03 - 1.08)	1.52e-07	5.40e-05	9.73e-01
RPS2;RNF151	cg18279742	rs758335	-0.71	0.07	1.07 (1.04 - 1.09)	2.47e-07	7.75e-05	9.73e-01
KLHL30	cg22741244	rs12618952	-0.21	-0.14	0.87 (0.81 - 0.94)	4.00e-04	2.22e-02	9.75e-01
CEP68	cg05010058	rs2723064	0.46	-0.14	0.87 (0.84 - 0.90)	2.55e-15	1.36e-11	9.91e-01
SNHG9;SNORA64;SNORA78;RPS2	cg00991848	rs397460	-0.46	0.10	1.10 (1.06 - 1.15)	1.36e-06	3.81e-04	9.94e-01

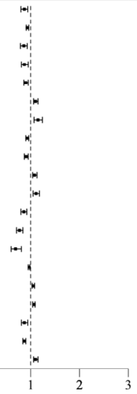

Figure S2. Mendelian randomization results for the association between primary cilia gene methylations and AF risk (PP.H4 > 0.8). The figure represents the effect size (β) of a variant on DNA methylation, with β > 0 indicating a positive association and β < 0 indicating a negative association. Odds ratios were calculated based on the expectation of the causal estimate (β coefficient).

Figure S3

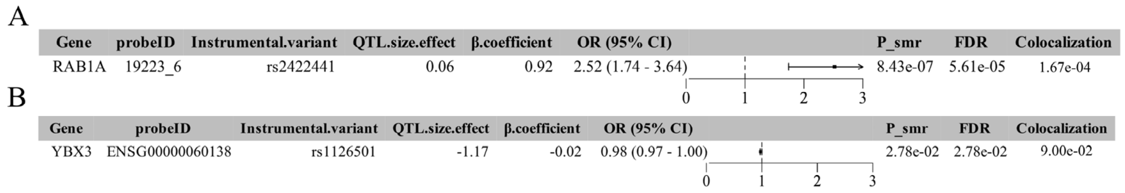

Figure S3. Mendelian randomization results for the association between the expression of primary cilia-related proteins and AF risk. (A) Blood sets (decode), (B) LAA tissue sets. The figure represents the effect size (β) of each variant on protein abundance, where β > 0 indicates a positive association and β < 0 indicates a negative association. Odds ratios were calculated based on the expectation of the causal estimate (β coefficient).

Figure S4

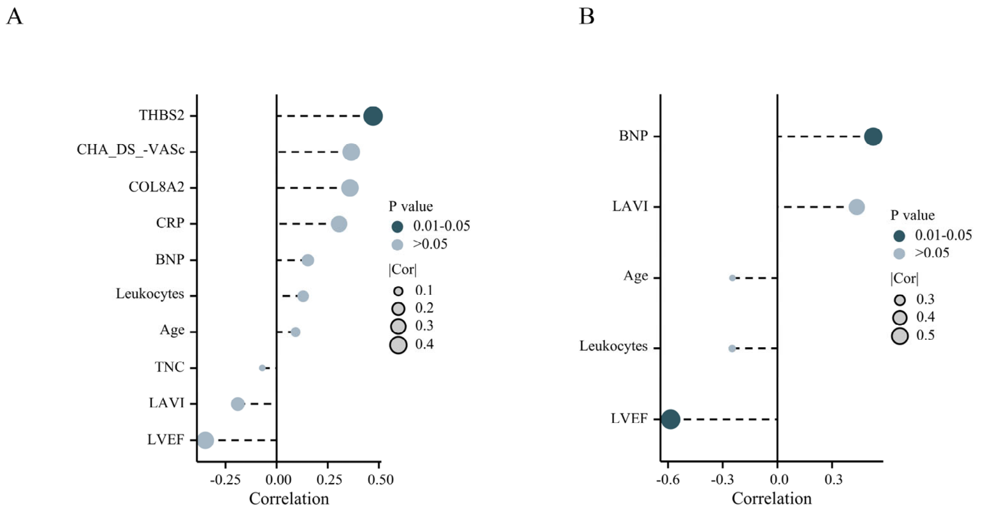

Figure S4. Clinical correlations of CEP68 (complete-case sensitivity analysis). (A) Correlation analysis between CEP68 and clinical indicators in 22 patients without AF using complete-case data. (B) Correlation analysis between CEP68 and clinical indicators in 19 patients with persistent AF using complete-case data. Abbreviations: AF, atrial fibrillation; BNP, B-type natriuretic peptide; CHA_2DS_2-VASc, Congestive heart failure, Hypertension, Age ≥75 (2 points), Diabetes mellitus, Stroke/TIA/thromboembolism (2 points), Vascular disease, Age 65–74, Sex category (female); COL8A2, collagen type VIII alpha 2; CRP, C-reactive protein;

LAVI, left atrial volume index; LVEF, left ventricular ejection fraction; TNC, tenascin-C; THBS2, thrombospondin-2.
